# Incidence of Childhood Leukemia in Iraq, 2000-2019

**DOI:** 10.31557/APJCP.2021.22.11.3663

**Published:** 2021-11

**Authors:** Muzahem M Y AL-Hashimi

**Affiliations:** *Department of Statistics and Informatics, College of Computer Science & Mathematics, University of Mosul, Mosul, Iraq. *

**Keywords:** Childhood leukemia, incidence, poisson regression, time trends

## Abstract

**Background:**

Leukemia is a major concern for children worldwide. Around 30% of malignancies in children (ages 0–14) are caused by leukemia.

**Objective:**

This study aims to explore the time trends in the incidence of childhood leukemia (aged 0-14 years) in Iraq between 2000 and 2019.

**Methods:**

Poisson regression with a log link function was used to analyze the long-term trends of incidence related to childhood leukemia cancer based on published data from the Iraqi cancer registry between 2000 and 2019. Annual estimates of the population, by 5-year age groups and by gender obtained from the United Nations, population Division.

**Results:**

A total of 8,570 cases of leukemia children in Iraq between 2000 and 2019 were recorded, the boys to girl ratio were 1.32 to 1. The most diagnosed type of leukemia was Acute lymphoblastic leukemia, accounting for about 33.56%, followed by Leukemia Not specify (NOS) (17.3%) with a relatively equal proportion of stated instances between boys and girls in these subsets. The age-standardized incidence rates, aged 0-14 years, from 2000-2019 were 3.45/100,000 for both genders. The Joinpoint regression ASRs analysis of childhood leukemia from 2000-2019 among 0 –14 age group for both genders indicate that there was an overall significant increasing trend at 1.23% per year, while no one joinpoint was identified during the entire study period. Among boys, there was an overall insignificant increasing trend at 0.77% per year. Among girls, there was an overall significant increasing trend at 1.93% per year, while one joinpoint was identified during the entire study period.

**Conclusions:**

The overall (both genders) incidence rate of childhood leukemia has been increasing significantly in Iraq. The test for trends was insignificant among boys, while it was significant among girls. The increasing trend of leukemia requires further epidemiological studies to describe incidence by geography in Iraq.

## Introduction

Leukemia is the most common cancer in children and adolescents worldwide, followed by brain and central nervous system malignancies (Park et al., 2016). Around 30% of malignancies in children (ages 0–14) and 0.10 of malignancies in adolescents (ages 15–19) are caused by leukemia (Parkin et al., 2002). Acute lymphocytic leukemia (ALL) is the most common subtype (Navarro et al., 2017). The underlying causes of childhood leukemia are not well known, (Namayandeh et al., 2020; Miranda et al., 2018), however, some risk factors are implicated with the development of cancer. Many studies showed that a combination of genetic risk factors and environmental were associated with the development of this malignancy (Filippini et al., 2015).

A recent report on the incidence of childhood leukemia in the United States suggested that Leukemia accounts for about 27% of childhood cancers before the age of 15 years and 0.35% before the age of 20 (Ward et al., 2014), 32.3% before the age of 15 in Canada (Lin et al., 2018), 26.5% before the age of 19 in Denmark (Grabas et al., 2020), 30% in Ireland (Stack et al., 2007) to 29.7% in Sweden (0-14) (Dreifaldt et al., 2004), and 34.5% in Shanghai, China (Bao et al., 2009). 

Between 2000 and 2019, cancers in children of both genders, aged 0-19 years, account for 8.97%, of all new cancer diagnoses in Iraq, there were 35411 childhood cancers, the most common being leukemia (10499) (29.65%), on average, 524 children are diagnosed with leukemia each year. For the years from 2000-2004, leukemia represented (2088) 30.94% of all cancer cases occurring among children (0-19 years of age), decreasing slightly (2174) 30.50% during the period (2005-2009), increasing to (3020) 32.82% during the period (2010-2014), and decreasing to (3217) 25.60% during the period (2015-2019).

The Iraqi Cancer Registry Center was established in 1974 through close cooperation of the Ministry of Health and the Iraqi Cancer Society. It began operation in 1975 and was located in the Central Public Health Laboratory. It aggregates information from governmental and nongovernmental health facilities in all Iraqi provinces. It published a yearly report outlining the burden of malignant growth in Iraq concerning frequency, as indicated by age, gender, topography, morphology, and spatial distribution (Iraqi Cancer Registry). Well-trained staff was taught to copy certain items from hospital records, as well as from radiation and pathology departments by CanReg 4. The completed forms are forwarded to the Ministry of Health’s central registration every three months, where they are verified for accuracy and completeness, and a listing or a card is created for the registry for each reportable case. To avoid duplication, finished records are placed into the “Alphabetical Index.” All arriving forms are verified and filled up with the patient’s name, registry number, and the International Classification of Diseases for Oncology (ICD-O) code and histology is classified using the ICD-O System (Iraqi Cancer Registry).

Monitoring cancer incidence and time trends are one of the most important functions for cancer research and health care planning. This study aims to explore the time trends in the incidence of childhood leukemia (aged 0-14 years) in Iraq over a period (20 years) between 2000 and 2019.

## Materials and Methods

The data for cancer of all types of Leukemia (International Classification of Diseases for Oncology (ICD-O-3) code) between 1st January 2000 and 31st December 2019 were taken from the annual book series published from the Iraqi cancer registry/Ministry of Health. Annual estimates of the population, by 5-year age groups and by gender obtained from the United Nations, population division, department of economic and social affairs (http://esa.un.org/wpp/Excel-Data/population.htm). 

Age-specific incidence rates (ASIRs) for 5-year age groups; 0–4, 5-9, and 10–14 were computed.

The gender-specific and age-specific rate (per 100,000 population) were computed using the following formula (Chen et al., 2014):

= (cases in a specific age group)/(population in the age group)×100,000

Age-standardized incidence rates (ASRs) using the world standard population of childhood Leukemia aged 0-14 years were computed for individual years, by 5-year period, and whole study period. The ASRs (per 100,000 population) were computed using the following formula:

$\mathrm{ASR}=\frac{\sum_{i=1}^{n}a_{i}w_{i}}{\sum_{i=1}^{n}w_{i}}\times 100$

(a_i_, where i denotes the ith age class), and the number of peoples (or weight) (w_i_) in age subgroup i of the selected reference standard population (Li et al., 2019). 

To find the best fit model according to age groups and gender, Joinpoint regression analysis software version 4.9.0.0. was applied for the annual percentage change estimation with the following: The covariate variable was the year of leukemia diagnosis and the response variable was the ASR, and the ASIRs for the incidence of childhood leukemia with log transformation. Homoscedasticity and uncorrelated error model was assumed in the calculation. The number of joinpoints was set to 0 as a minimum and 4 as a maximum. For all analyses, the significance level was set at p ≤ 5%.

## Results

**Figure 1:**
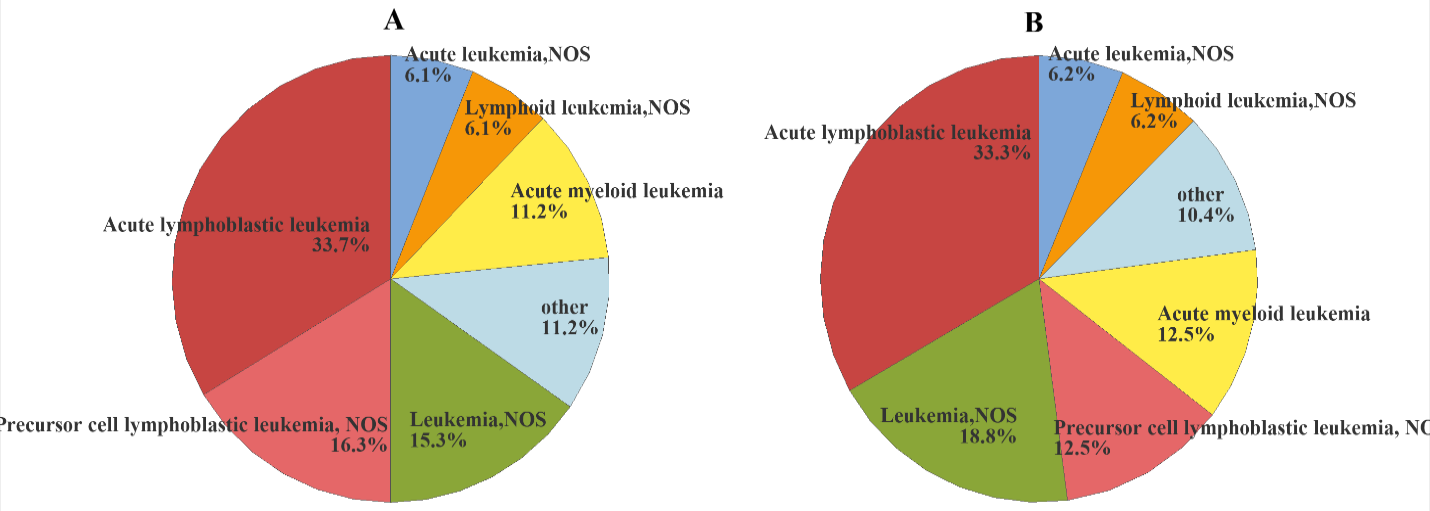
Morphological Distribution Childhood Leukemia, 2000-2019, Iraq. Morphological distribution among boys (A), Morphological distribution among girls (B)

Eight thousand five hundred and seventy cases of leukemia children (0-14 years of age) in Iraq between January 1st, 2000 and December 31 2019 were documented, for an average of 429 new cases per year. leukemia accounts for 32.96% of all cancers diagnosed in children under 15 years of age. Acute lymphoblastic leukemia (ALL) was the most common leukemia type, accounting for about (2876) 33.56% of childhood leukemia in Iraq, Leukemia Not specify (NOS), Precursor cell lymphoblastic leukemia Not specify (NOS), Acute myeloid leukemia, Lymphoid leukemia Not specify (NOS), and Acute leukemia Not specify (NOS) which include (1539) 17.96%, (1268) 14.80%, (1101) 12.85%, (562) 6.56%, and (551) 6.43% of total 21 types of leukemia, respectively. These values were (1617) 33.2%, (740) 15.2%, (794) 16.3%, (565) 11.6%, (292) 6.0%, and (312) 6.4% for boys, and (1184) 32.0%, (695) 18.8%, (477) 12.9%, (470) 12.7%, (248) 6.7%, and (226) 6.1% for girls, respectively (Figure 1). 

Leukemia was more common in boys than girls, the boys to girls ratio was 1.32 to 1, and there were some variations in incidence between age groups. The infection was highest among the age group (0-4) (3596) (41.96%), followed by children among age group (5-9) (2828) (33.00%), and lowest for the age group (10-14) (2146) (25.04%). According to age groups, the highest leukemia ASIR for both genders were estimated among the age group of 0–4 years (3.88 per 100,000), followed by age group (5-9) (3.41 per 100,000), and lowest for the age group (10-14) (2.90 per 100,000). 

The age-standardized incidence rates, aged 0-14 years, during the period 2000-2019 were 3.45 per 100,000 for both genders (the annual ASR ranged from 2.56 per 100,000 in 2008 to 4.58 per 100,000 in 2010), and 3.81 per 100,000 for boys (the annual ASR ranged from 2.68 per 100,000 in 2009 to 4.556 per 100,000 in 2004), and 3.06 per 100,000 for girls (the annual ASR ranged from 1.92 per 100,000 in 2002 to 4.03 per 100,000 in 2010). 

Table 1 illustrates the ASR of childhood leukemia (0-14) in Iraq by 5-year period. The ASR in both genders raised from 3.16 per 100,000 during the period 2000-2004 to 3.62 during the period 2015-2019. The average number of childhood leukemia diagnosed each year over these two periods increased from 23.11 to 26.49. Among boys, the average number of childhood leukemia diagnosed each year over the same period increased from 24.16 to 26.20, while among girls increased from 21.62 to 26.88.

Detailed investigation of the data shows fluctuations from period to period. As illustrated in Table 1, the ASR of both genders of the last two 5-year periods seemed insignificantly higher than that of the reference period (2000-2004). These rates were respectively 1.22 and 1.16 times higher than that of the reference period (2000-2004). Among boys, the ASR of the last two 5-year periods appeared insignificantly higher than that of the reference period by 1.12 and 1.08 times, while for girls, the ASR appeared insignificantly higher than that of the reference period by 1.37 and 1.24 times, respectively.

**Figure 2:**
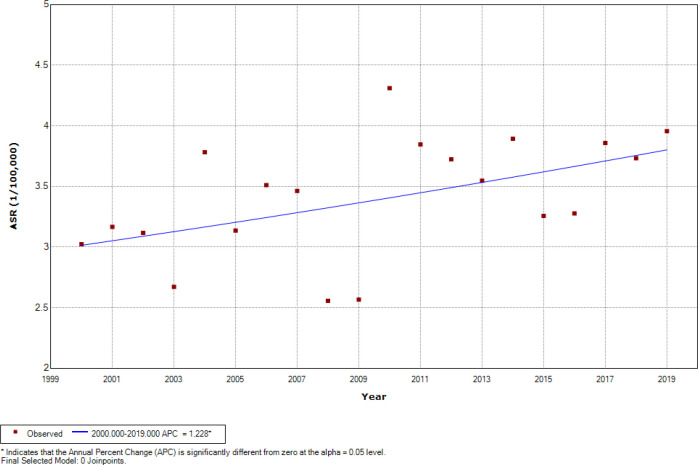
Significant Joinpoints in Trends of Childhood Leukemia ASR (Per 100,000 Children-Years) for Age (0–14 Years) among Both Genders in Iraq, 2000–2019

**Figure 3:**
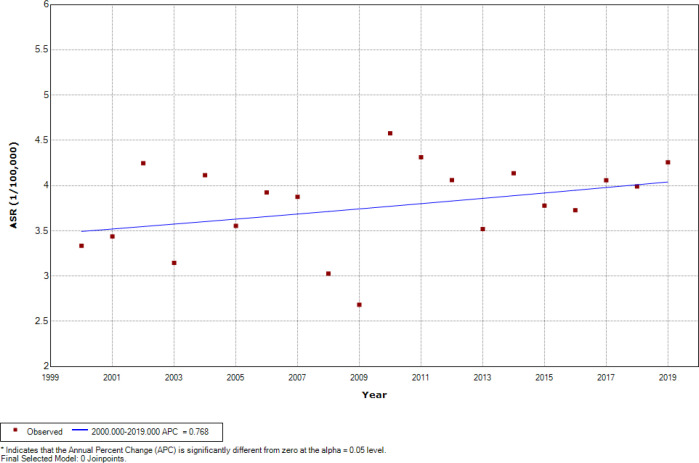
Insignificant Joinpoints in Trends of Childhood Leukemia ASR (Per 100,000 Children-Years) for Age (0–14 Years) among Boys in Iraq, 2000–2019

**Figure 4:**
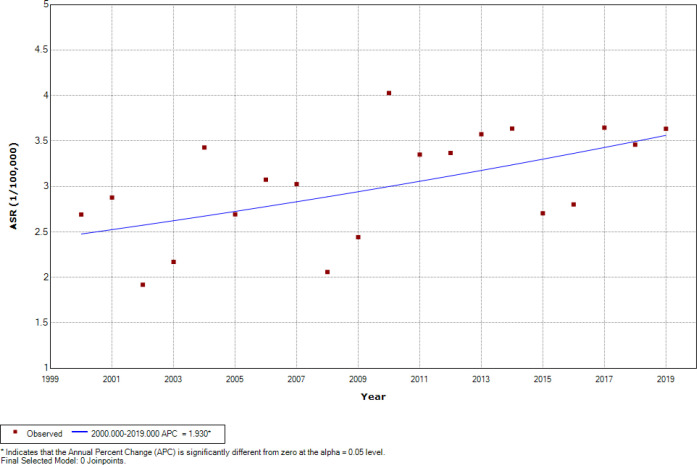
Significant Joinpoints in Trends of Childhood Leukemia ASR (Per 100,000 Children-Years) for Age (0–14 Years) among Girls in Iraq, 2000–2019

**Figure 5:**
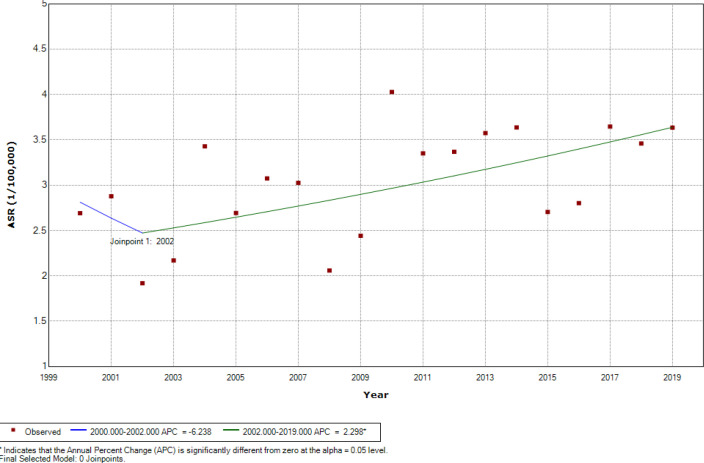
Joinpoints in Trends of Childhood Leukemia ASR (Per 100,000 Children-Years) for Age (0–14 years) among Girls in Iraq, 2000–2019. The point in time selected where trends significantly change direction at any given year

Figure 2 represents the findings of Joinpoint regression ASRs analysis of childhood leukemia during the period 2000-2019 for 0 –14 age group among both genders, there was an overall significant increasing trend at 1.23% per year (CI=0.14 to 2.33), while no one joinpoint was identified during the entire study period. Among boys, there was an overall insignificant increasing trend at 0.77% per year (CI=-0.30 to 1.84) (Figure 3). Among girls, there was an overall significant increasing trend at 1.93% per year (CI=-0.45 to 3.43) (Figure 4), while one joinpoint was identified during the entire study period. The ASRs decreased insignificantly until 2002 then increased significantly (from -6.24 to 2.30% per year) from 2002 to 2019 (Figure 5).

The results of Joinpoint regression of childhood leukemia from 2000 to 2019 for both genders according to age groups are illustrated in Table 2. In the 0–4 age group, the ASIR increased significantly at 2.47% (CI=1.03 to 3.93) per year. In the 5-9 age group, the ASIR increased insignificantly at 0.53% (CI=-0.53 to 1.59) per year, and in the 10 –14 age group, the ASIR increased insignificantly at 0.03% (CI=-1.39 to 1.48) per year.

**Table 1:** The Age-Standardized Incidence Rate of Childhood Leukemia by Gender in Iraq during the Period 2000 to 2019 with the Results of the Time Trend Analysis: APC Related to Each 5-Year Period

Period	Demographic data	Number (%)	ASR (95% CI)	APC (95% CI)	p value
2000-2004	0-14 both genders	1675 (19.54)	3.16 (2.50-3.76)	Ref.	
2005-2009		1781 (20.78)	3.08 (2.35-3.53)	-2.53 (-11.60, 5.90)	0.97
2010-2014		2499 (29.16)	3.86 (2.94-4.04)	22.15 (1.40, 32.10)	0.79
2015-2019		2615 (30.51)	3.62 (2.79-4.19)	14.56 (-2.90, 23.40)	0.86
2000-2004	0-14 boys	999 (20.51)	3.66 (2.91-4.37)	Ref.	0.11
2005-2009		1028 (21.10)	3.40 (2.66-4.00)	-7.11 (-18.80, 5.50)	0.92
2010-2014		1371 (28.15)	4.11 (3.17-4.75)	12.30 (-2.20, 21.50)	0.87
2015-2019		1473 (30.24)	3.97 (3.12-4.68)	8.47 (-3.80, 16.50)	0.91
2000-2004	0-14 girls	676 (18.28)	2.62 (2.07-3.11)	Ref.	0.57
2005-2009		753 (20.36)	2.65 (2.02-3.04)	1.15 (-5.60, 9.50)	0.99
2010-2014		1128 (30.49)	3.59 (2.69-4.03)	37.03 (8.80, 56.60)	0.7
2015-2019		1142 (30.87)	3.26 (2.45-3.67)	24.43 (7.50, 47.10)	0.79

**Table 2:** The Age-Specific Incidence Rates (ASIRs) of Childhood Leukemia by Age Group in Iraq during the Period 2000 to 2019 with the Results of the Time Trend

Period	Demographic data	Number (%)	ASR (95% CI)	APC (95% CI)	p value
2000-2019	0-4	3596 (41.96)	3.88 (3.10-4.66)	2.47 (1.03, 3.93)	0.01
	5-9	2828 (33.00)	3.41 (2.73-4.09)	0.53 (-0.53, 1.59)	0.31
	10-14	2146 (25.04)	2.90 (2.32-3.48)	0.03 (-1.39, 1.48)	0.96

**Figure 6:**
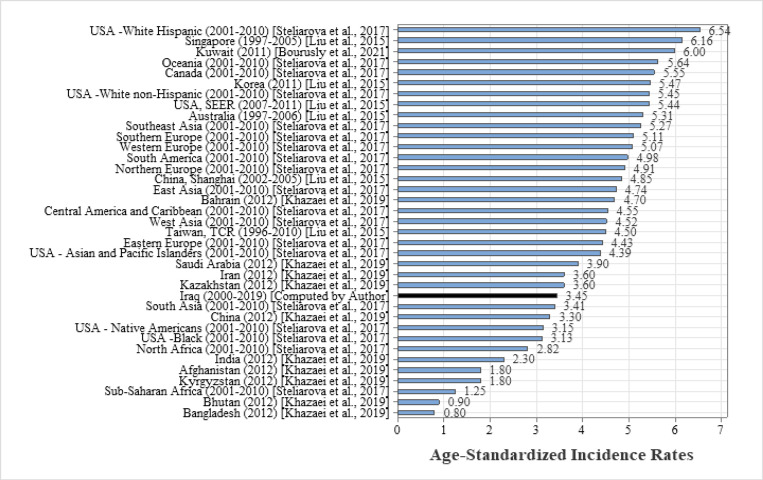
The Age-Standardized Incidence Rates of Childhood Leukemia (0 - 14 years) in Iraq and Selected Countries

**Figure 7:**
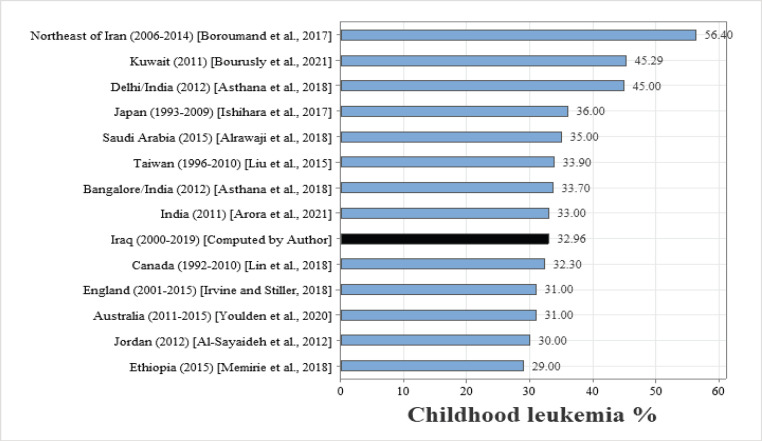
The Proportion of Childhood Leukemia (0 - 14 Years) in Iraq and Selected Countries

## Discussion

leukemia is the most common type of cancer in children aged 0–14 (Namayandeh et al., 2020; Fajardo et al., 1995; Fajardo et al., 1999; Ries et al., 2008). According to the results found by Namayandeh et al., (2020), the highest incidence of childhood leukemia in both genders in 2018 was recorded in Malaysia (8 per 100,000) and the Republic of Moldova (7.2 per 100,000). The lowest incidence appeared in Sub-Saharan Africa (3.40 per 100,000), followed by Bhutan with ASR (0.90 per 100,000) and Bangladesh (0.90 per 100,000).

Steliarova-Foucher et al., (2017) suggested various incidence rates of childhood leukemia. Furthermore, they demonstrated that the low incidence in low Human Development Index (HDI) countries was probably due to lower diagnosis.

This paper is the first to describe trend analyses of childhood leukemia in Iraq. This study aims to explore the time trends in the incidence of childhood leukemia (aged 0-14 years) in Iraq over a period (20 years) between 2000 and 2019. Data were available for this paper from the annual book series published by the Iraqi cancer registry/Ministry of Health. 

According to the results obtained from the studies on the prevalence of leukemia in some provinces in Iraq, the ASR of childhood leukemia (0-14 aged years) in Basrah from 2003 to 2007 was 5.45 per 100,000 (Alrudainy et al., 2009). Hagopian et al., (2010) found that the rates of childhood leukemia (0-14 aged year) in Basrah ranging between 15 cases (2.6 per 100,000 annual rates) in 1993 and 56 cases (6.9 per 100,000 annual rates) in 2007, reaching a peak of 97 cases in 2006 (12.2 per 100,000 annual rates). Khoshnaw et al., (2016) found that the rates of childhood leukemia (0-14 aged year) in Sulaymaniyah province is 2.97 per 100,000 in 2006 and it is 3.57 per 100,000 during the period from 2006 to 2014. Mjali et al., (2019) found that ALL was the most common kind of leukemia among the younger age groups in Kerbala, accounting for about 87.9% of leukemia cases in the age 10 years. 

Our results were similar to what has been reported in South Asia (ASR was 3.41 per 100,000 during the period 2001-2010), lower than that observed in the USA – White Hispanic, Oceania, Canada, and numerous Arab nations such as Kuwait, Jordan, Bahrain, Saudi Arabia. This rate was greater than that estimated in other nations, including the USA – Native Americans, USA – Black, China, and India (Figure 6). 

Leukemia was the more frequent type of childhood cancer in Iraq, accounting for 32.96% during 2000-2019, this result was similar to what has been reported in India (33.0% in 2011) (Arora et al., 2021). Less than Taiwan (33.9% during 1996–2010) (Liu et al., 2015), Saudi Arabia (35% in 2015) (Alrawaji et al., 2018), Japan (36.0% during 1993-2009) (Ishihara et al., 2017), Northeast of Iran (56.4% during 2006-2014) (Boroumand et al., 2017), Kuwait (45.29% in 2011) (Bourusly et al., 2021), Delhi/India (45% in 2012) (Asthana et al., 2018), Bangalore/India (33.7% in 2012) (Asthana et al., 2018), greater than Ethiopia (29% in 2015) (Memirie et al., 2018), Jordan (30% in 2012) (Al-Sayaideh et al., 2012), England (31% during 2001 to 2015) (Irvine and Stiller, 2018), Australia (31% during 2011 to 2015) (Youlden et al., 2020), and Canada (32.3% during 1992-2010) (Lin et al., 2018) (Figure 7).

A study from Kuwait reported a marginally significant increase in ASIRs among children (0–19 years) (APC = 2.5) between 1994 and 2014 (Akhtar et al., 2020). A study from Delhi, India documented an increasing trend among children (0–14 years) (APC = 4.58) between 1999 and 2014 (Malhotra et al., 2021), These estimates are substantially greater than the estimates reported in this study. In Canada, from 2002 to 2006, the (APC=3.0) showed a statistically insignificant increase (Mitra et al., 2012). In Italy (Piedmont), from 1967 to 2011, leukemia incidence decreased insignificant (APC = -0.6) (Isaevska et al., 2017). In China, The ASR for leukemia (APC=3.4) in boys (0-14) increased significantly from 2000 to 2015 and increased significantly (APC=2.0) in girls for the same period (Sun et al., 2020). In Europe, during the period from 1991 to 2010, there was a significantly increasing trend in leukemia incidence rates among children (0–14 years) (APC = 0.66) (Steliarova et al., 2018). Similarly, between 1975 and 2014, the age-adjusted incidence rate of all children leukemia types among all races in the United States increased significantly (AAPC = 1.7) (Howlader et al., 2017).

Leukemia is the most frequent malignant disease affecting children. Thus far, the etiology of childhood leukemia remains largely unknown. Few risk factors (genetic susceptibility, ionizing radiation which has been significantly linked to acute lymphoblastic leukemia or acute myeloid leukemia (Belson et al., 2007), infections, etc.) have been recognized, however, they seem to clarify just a little extent of cases. Considerably more uncertain is the role of other environmental risk factors, such as indoor and outdoor air pollution (Filippini et al., 2015). 

In recent years, advancements in diagnosis and improved reporting of cases may have contributed to an increase in childhood leukemia incidence in Iraq. However, it is beyond the aim of this paper to know the reasons for childhood leukemia in Iraq. The causes of childhood leukemia in Iraq are unclear, but they may be partly explained by differences in risk factors such as genetic susceptibility, ionizing radiation. The increasing trend of leukemia requires further epidemiological studies to describe incidence by geography in Iraq.

In conclusion, the overall (both genders) incidence rate of childhood leukemia has been increasing significantly in Iraq. The test for trends was insignificant among boys, while it was significant among girls. The increasing trend of leukemia requires further epidemiological studies to describe incidence by geography in Iraq.

## Author Contribution Statement

None.

## Availability of data

The paper was conducted based on the annual book series published by the Iraqi cancer registry/Ministry of Health. Iraqi Cancer Board each year published detailed statistical data on cancer in Iraq. Annual estimates of the population, by 5-year age groups and by gender obtained from the United Nations, Population Division, Department of Economic and Social Affairs.

## Conflict of interest

I declare that I have no conflicts of interest to disclose regarding this manuscript. 

## References

[B1] Akhtar S, Al-Abkal J, Al-Shammari A (2020). Childhood leukaemia incidence and trends in a Middle Eastern country during 1980–2014: a population-based study. Cancer Causes Control.

[B2] Alrawaji A, Alshahrani Z, Alzahrani W (2018). Cancer Incidence Report Saudi Arabia 2015.

[B3] Alrudainy LA, Salih HM, Aldorky M (2009). Incidence and pattern of childhood leukaemia in Basrah, Iraq during 2003-2007. Iran J Blood Cancer.

[B5] Arora RS, Bagai P, Bhakta N (2021). Estimated national and state level incidence of childhood and adolescent cancer in India. Indian Pediatr.

[B6] Asthana S, Labani S, Mehrana S (2018). Incidence of childhood leukemia and lymphoma in India. Pediatr Hematol Oncol J.

[B7] Bao PP, Zheng Y, Wang CF (2009). Time trends and characteristics of childhood cancer among children age 0–14 in Shanghai. Pediatr. Blood Cancer.

[B8] Belson M, Kingsley B, Holmes A (2007). Risk factors for acute leukemia in children: a review. Environ. Health Perspect.

[B9] Boroumand H, Moshki M, Khajavi A (2017). Epidemiology of childhood cancer in northeast of Iran. Pediatr. Hematol Oncol J.

[B10] Bourusly MJ, Burahma MH, Khalifa N (2021). Trends in childhood cancer in Kuwait: Data From the 2004-2017 Registry. Cureus.

[B11] Chen W, Zheng R, Zhang S (2014). Report of cancer incidence and mortality in China, 2010. Ann Transl Med.

[B12] Dreifaldt AC, Carlberg M, Hardell L (2004). Increasing incidence rates of childhood malignant diseases in Sweden during the period 1960–1998. Eur J Cancer.

[B13] Fajardo-Gutierrez A, Mejia-Arangure JM, Hernandez-Cruz L (1999). Descriptive epidemiology of malignant tumors in children. Rev Panam Salud Publica.

[B14] Fajardo-Gutierrez A, Mejía-Aranguré M, Gómez-Delgado A (1995). Epidemiología de las neoplasias malignas en ni-os residentes del Distrito Federal (1982-1991) [In Spanish]. Bol Med Hosp Infant Mex.

[B15] Filippini T, Heck JE, Malagoli C (2015). A review and meta-analysis of outdoor air pollution and risk of childhood leukemia. J Environ Sci Health Part C.

[B16] Grabas MR, Kjaer SK, Frederiksen MH (2020). Incidence and time trends of childhood cancer in Denmark, 1943–2014. Acta Oncol.

[B17] Hagopian A, Lafta R, Hassan J (2010). Trends in childhood leukemia in Basrah, Iraq, 1993–2007. Am J Public Health.

[B18] Howlader NN, Noone AM, Krapcho M (2017). SEER cancer statistics review, 1975-2014, National Cancer Institute. Bethesda.

[B21] Isaevska E, Manasievska M, Alessi D (2017). Cancer incidence rates and trends among children and adolescents in Piedmont, 1967–2011. PLoS One.

[B22] Ishihara H, Ohno Y, Fujii M (2017). Epidemiological analysis of childhood cancer in Japan based on population-based cancer registries, 1993–2009. Jpn J Clin Oncol.

[B23] Khazaei Z, Goodarzi E, Adineh HA (2019). Epidemiology, incidence, and mortality of leukemia in children early infancy to 14 years old of age in South-Central Asia: A Global Ecological Study. J Compr Ped.

[B24] Khoshnaw N, Mohammed HA, Abdullah DA (2016). Patterns of cancer in Kurdistan-results of eight years cancer registration in Sulaymaniyah Province-Kurdistan-Iraq. Asian Pac J Cancer Prev.

[B25] Li NA, Deng Y, Zhou L (2019). Global burden of breast cancer and attributable risk factors in 195 countries and territories, from 1990 to 2017: results from the Global Burden of Disease Study 2017. J Hematol Oncol.

[B26] Lin X, Jay O, Howard M (2018). Childhood cancer incidence in Canada: demographic and geographic variation of temporal trends (1992–2010). Health Promot Chronic Dis Prev Can.

[B27] Liu YL, Lo WC, Chiang CJ (2015). Incidence of cancer in children aged 0–14 years in Taiwan, 1996–2010. Cancer Epidemiol.

[B28] Malhotra RK, Manoharan N, Nair O (2021). Patterns and trends of childhood cancer incidence (0–14 years) in Delhi, India: 1990–2014. Indian Pediatr.

[B29] Memirie ST, Habtemariam MK, Asefa M (2018). Estimates of cancer incidence in Ethiopia in 2015 using population-based registry data. J Glob Onco.

[B30] Miranda-Filho A, Piñeros M, Ferlay J (2018). Epidemiological patterns of leukaemia in 184 countries: a population-based study. Lancet Haematol.

[B31] Mitra D, Shaw AK, Hutchings K (2012). Trends in incidence of childhood cancer in Canada, 1992-2006. Chronic Dis Inj Canada.

[B32] Mjali A, Al-Shammari HH, Abbas NT (2019). Leukemia epidemiology in Karbala province of Iraq. Asian Pac J Cancer Care.

[B33] Namayandeh SM, Khazaei Z, Najafi ML (2020). GLOBAL leukemia in children 0-14 statistics 2018, incidence and mortality and human development index (HDI): GLOBOCAN Sources and Methods. Asian Pac J Cancer Prev.

[B34] Navarro SM, Matcuk GR, Patel DB (2017). Musculoskeletal imaging findings of hematologic malignancies. Radiographics.

[B35] Park HJ, Moon EK, Yoon JY (2016). Incidence and survival of childhood cancer in Korea. Cancer Res Treat.

[B36] Parkin DM, Whelan SL, Ferlay J (2002). Cancer incidence in five continents volume VIII. IARC Sci Publications.

[B38] Stack M, Walsh PM, Comber H (2007). Childhood cancer in Ireland: a population-based study. Arch Dis Child.

[B39] Steliarova-Foucher E, Colombet M, Ries LA (2017). International incidence of childhood cancer, 2001–10: a population-based registry study. Lancet Oncol.

[B40] Steliarova-Foucher E, Fidler MM, Colombet M (2018). Changing geographical patterns and trends in cancer incidence in children and adolescents in Europe, 1991–2010 (Automated Childhood Cancer Information System): a population-based study. Lancet Oncol.

[B41] Ward E, DeSantis C, Robbins A (2014). Childhood and adolescent cancer statistics, 2014. CA Cancer J Clin.

[B42] Youlden DR, Baade PD, Green AC (2020). The incidence of childhood cancer in Australia, 1983–2015, and projections to 2035. Med J Aust.

